# Complex economic decisions from simple neurocognitive processes: the role of interactive attention

**DOI:** 10.1098/rspb.2022.1593

**Published:** 2023-02-08

**Authors:** Lisheng He, Sudeep Bhatia

**Affiliations:** ^1^ SILC Business School, Shanghai University, Shanghai, People's Republic of China; ^2^ Department of Psychology, University of Pennsylvania, Philadelphia, PA, USA

**Keywords:** decision making, attention, risk, neurocognitive processes, computational modelling

## Abstract

Neurocognitive theories of value-based choice propose that people additively accumulate choice attributes when making decisions. These theories cannot explain the emergence of complex multiplicative preferences such as those assumed by prospect theory and other economic models. We investigate an *interactive attention* mechanism, according to which attention to attributes (like payoffs) depends on other attributes (like probabilities) attended to previously. We formalize this mechanism using a Markov attention model combined with an accumulator decision process, and test our model on eye-tracking and mouse-tracking data in risky choice. Our tests show that interactive attention is necessary to make good choices, that most participants display interactive attention and that allowing for interactive attention in accumulation-based decision models improves their predictions. By equipping established decision models with sophisticated attentional dynamics, we extend these models to describe complex economic choice, and in the process, we unify two prominent theoretical approaches to studying value-based decision making.

## 1. Introduction

There are two major theoretical approaches to modelling value-based decision making. The first, common in economics and related branches of social science, proposes that people maximize utility functions, which can be derived based on normative axioms and their observed violations [1–6]. Such utility functions are frequently used for economic forecasts, policy prescriptions, and analysis of social and organizational phenomena. The second approach, common in fields like psychology and neuroscience, proposes that people engage in information sampling and evaluation processes, which can be studied using laboratory-based attention measures (such as eye-tracking or mouse-tracking), as well as choice and response time data [7–11]. These processes are the products of mental algorithms and can be interpreted in terms of underlying cognitive and neural mechanisms.

Unifying these two theoretical traditions by showing how behaviour predicted by standard utility functions can be generated by cognitively and neurally plausible information sampling processes has been the focus of considerable interdisciplinary research [12–18]. Yet major challenges remain. At the heart of the problem is the fact that most standard utility functions propose complex multiplicative interactions between the attributes of choice options. For example, expected value (EV) maximization, the simplest economic model of risky choice, assumes that payoffs are multiplied against corresponding probabilities, and that these probability-weighted payoffs are aggregated into a utility value for the gamble. Expected utility theory, cumulative prospect theory and other subjective expected utility theories (SEUT) retain the multiplicative structure of EV, while modifying the subjective-value function and probability weights, respectively [1,2]. Models such as exponential discounting and hyperbolic discounting assume a similar set of interactions between payoffs and time delays in intertemporal choice, and interactions between the attributes of options are also at play in many multi-attribute, social and strategic choice settings [3–5].

Most cognitive and neurocognitive models of decision making, by contrast, propose that decision makers sample one piece of information at a given point in time, and accumulate the sampled value of this information sequentially and additively into a decision variable. Choice is made when this decision variable crosses a threshold [8,9]. In some accumulator models, changes to the decision variable are determined by attention to choice attributes, whereas in others, changes are determined by attention to choice items [11,19–26]. Visual-fixation and mouse-movement data allow researchers to observe these attentional inputs, and thus specify the rate of accumulation at each time point during deliberation. Accumulator models have been shown to provide a good account of the processes underlying simple preferential choice. However, it is unclear how these approaches could be applied to more complex decisions, which are the focus of much of the research in economics and social sciences. In risky decisions, for example, decision makers are shown the payoffs and probabilities of two or more gambles and are allowed to obtain information about these gambles through visual fixations on (or mouse movements to) the individual payoffs and probabilities [27–35]. They may, for example, attend to one of the payoffs of the first gamble, before looking at one of the probabilities of the second gamble. It is likely that such an attentional process influences the accumulation of decision variables, and determines choice and response time [10,22,23]. However, in order to mimic the desirable properties of multiplicative economic theories, accumulator models need to involve the multiplicative, rather than additive, accumulation of payoffs and probabilities.

The difficulty of multiplicatively accumulating sequentially sampled attributes in decision making can be seen as a type of *binding problem*, one that is closely related to the problem of segregating and combining the features of objects in perceptual tasks [36–38]. In such tasks, models that aggregate attributes additively would confuse a red circle and a blue square with a blue circle and a red square. To solve this binding problem, vision researchers have proposed many solutions, some of which rely on the biased allocation of attention to the location of the object [36]. In this paper, we explore a similar (attentional) solution to the problem of how neurobiologically plausible processes generate the multiplicative utility functions of economic theory.

It may be argued that people do not engage in such a binding process, and that behaviour is better described by simple additive models (in which choice is a function of the weighted sum of payoffs and probabilities) than by multiplicative SEUT. Additive models of risk were proposed by psychologists many decades ago [39,40], and have also been reconsidered by more recent research [11,41]. These models are suitable for simple risky choice tasks with single-branch gambles offering only one non-zero payoff with a positive probability (e.g. $100 with probability 0.5, and $0 otherwise). In such tasks, multiplicative utility functions can be log-transformed into additive functions without altering preference orderings. However, additive models are unable to make reasonable predictions in multi-branch gambles, which involve multiple payoffs, each with their own probabilities.

As an example, consider the choice between a gamble *X* offering a payoff of $100 with probability 99% and a payoff of $1 with probability 1%, and a gamble *Y* offering a payoff of $100 with probability 1% and a payoff of $1 with probability 99%. Since *X* strongly dominates *Y*, participants always choose *X*. SEUT models, like prospect theory, which multiply (subjective) payoffs against corresponding (subjective) probabilities, have no difficulty generating this preference. Additive models, by contrast, consider the payoffs and probabilities separately. These models know that both of the gambles offer payoffs of $100 and $1, and that both have probabilities of 99% and 1%, but cannot multiply branch-specific payoffs and probabilities to develop a preference for *X*. In other words, the additive preference accumulation models are unable to bind the branches of multi-branch risky gambles in order to appropriately determine their value.

We examine a solution to the binding problem in decision making, in order to relate accumulation-based cognitive and neurocognitive models of decision processes to the types of multiplicative SEUT functions studied in economics. As with established models in psychology and neuroscience, our solution assumes that preferences accumulate dynamically with additive increments determined by inputs from attentional processes. However, we propose that attentional processes display nuanced temporal dynamics, so that attending to high probabilities increases the likelihood of attending to the payoffs in the same gamble branch, and vice versa. This *interactive attention* process implies that the inputs into the decision variable are not independent over time; rather the rate of accumulation at a given point of time depends on what was sampled previously. Crucially, interactive attention causes payoffs that occur with high probabilities (and probabilities that are associated with high payoffs) to be more likely to be sampled and thus play a larger role in the accumulation processes. For the choice presented in the previous paragraph, this implies that there would be higher attention to a payoff of $100 when it occurs with a probability of 99% (as in gamble *X*) than when it occurs with a probability of 1% (as in gamble *Y*). This would guide accumulating preferences towards *X* and result in a choice of *X* over *Y*.

**Figure 1.**
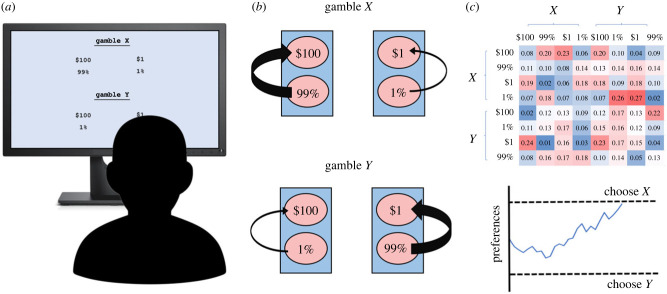
Experimental paradigm, interactive attention mechanism and computational model. (*a*) Participants are shown multi-branch gambles on a computer screen and are asked to choose between them. (*b*) They make this decision by attending to the probabilities and payoffs involved in the gambles. Attention is interactive, so that attending to a high probability (e.g. 99% in gamble *X*) increases the probability of attending to the corresponding branch payoff (e.g. $100 in gamble *X*). (*c*) The full attention process is described by a Markov model, in which the transition matrix (top panel) depends on the underlying payoffs and probabilities. Decision makers additively aggregate sampled probabilities and payoffs into a decision variable that evolves over time (bottom panel). Decisions are made when this variable crosses a decision threshold.

Some cognitive models of risky choice have assumed that attention to payoffs depends on their underlying probabilities [11,26,42]. Additionally, researchers have shown that a feedback effect, according to which high attribute values direct attention to other attributes in the same option, is common in multiattribute choice and multicue judgement [23,43–46]. This is referred to as the *attraction search* effect, and is similar to interactive attention effect examined in the current paper, except that the attraction search effect involves an attentional bias that is directed towards all attributes of the option, whereas the interactive attention effect involves an attentional bias that is directed towards only the attributes in the same branch as the high-valued attribute. In this paper, we formalize the interactive attention effect using a Markov model that specifies attention dynamics, combined with an accumulator process that aggregates preferences based on attention [10,11,19–26]. We test the effect by fitting data from two new experiments and four existing experiments consisting of both eye-tracking and mouse-tracking attention measures in multi-branch risky choice [27,29]. Our results indicate that our attention model can describe both the attentional dynamics and emergent preferences of individuals in these datasets. The interactive attention mechanism can also generate the types of multiplicative preferences proposed by mainstream economic theories (e.g. a preference for *X* over *Y* in the previous paragraph), while retaining the additive accumulation assumptions of established cognitive and neurocognitive models of choice (see figure 1 for an illustration of our experimental paradigm and model).

## 2. Methods

### (a) Experimental data

We ran two new risky choice experiments in laboratory using the MouseLab paradigm (exp. 1: *N* = 54, *M*_age_ = 20.2 ± 1.1, 43% female; exp. 2: *N* = 49, *M*_age_ = 20.0 ± 1.1, 55% female). In both experiments, each participant made 32 binary choices between five-branch gambles. The payoffs were randomly sampled from the uniform distribution between $0 and $10 (integers only). The five probabilities in each gamble were generated as follows. The first probability, *p*_1_, was a random number between 0 and 1; the second probability, *p*_2_, was a random draw between 0 and 1– *p*_1_; and so on. Finally, the fifth probability was 1 – *p*_1_ – *p*_2_ – *p*_3_ – *p*_4_. The five probabilities were then randomly paired with the five payoffs within each gamble. The full list of choice problems can be found in electronic supplementary material, table S1.

At the beginning of each trial, the payoffs and probabilities were hidden behind boxes. Participants clicked on the boxes to reveal a payoff or a probability of each gamble. Each time they clicked on a box, the payoff or probability underneath appeared while the payoff or probability in the previous click was simultaneously hidden. Participants could search as many times as they wanted, until they chose one of the two gambles with a key press. The two experiments differed only in the way in which the payoffs and probabilities were presented (see electronic supplementary material, figure S1). In exp. 1, payoffs and the associated probabilities of each gamble were presented side by side. By contrast, in exp. 2, the lists of payoffs from the two gambles were presented side by side on the left-hand side of the screen and the lists of probabilities from the two gambles were presented side by side on the right-hand side of the screen. This was done to ensure that observed interactive attention effects were not a product of the placement of the choice attributes. In data analysis, we excluded the trials where the participant opened less than two boxes, or no choice data were recorded in that trial (i.e. 4.49% of all the trials in the two experiments).

We also reanalysed two published studies of multi-branch risky choice with information acquisition data (as in figure 1*a*), one by Pachur *et al*. [27] and the other by Fiedler & Glöckner [29]. Pachur *et al*. had two mouse-tracking experiments, involving 90 participants making risky choices in two separate sessions, with a break of at least one week between sessions. We treated the two sessions as two separate experiments. Both experiments included 90 two-branch risky choices. The choice problems involved gains, losses and mixed gambles. See electronic supplementary material, table S2 for the list of choice items. Note that Pachur *et al*. had another experiment consisting of three between-subject conditions. We did not use that experiment for our analysis, as that experiment manipulated attention (and we were interested only in natural attention dynamics).

Fiedler and Glöckner had two eye-tracking experiments, one with 21 participants, and the other with 36 participants. In both experiments, each participant made 50 two-branch risky choices in the gain domain. In exp. 1, 40 of the 50 choice problems involved pairs of gambles with the same or similar EVs. In exp. 2, the absolute EV difference between gambles and the mean EV was systematically controlled such that they were uncorrelated across choice problems. See electronic supplementary material, table S3 for the details of choice problems. Our reanalysis was based on the cleaned data made public by the original authors.

### (b) Markov attention models

Our major innovation is in specifying the dynamics of the attentional process. We assume that sequences of attentional states are not independent. Rather, when decision makers switch attentional states, the probability of the subsequent attentional state depends on the previous attentional state. These dynamics can be described by a Markov process. In the case of a multi-branch risky choice between gambles *X* offering *N* payoffs, and *Y* offering *M* payoffs, there are a total of 2 *N* + 2 *M* attentional states, one for each distinct probability and payoff involved. Thus, the Markov process involves an (2 *N* + 2 *M*) × (2 *N* + 2 *M*) transition matrix. In our experiments, the Markov attention models predict switches between the 20 attentional states (in the five-branch experiments) or eight attentional states (in the two-branch experiments) (denoted by S) using transition probabilities $\mathrm{Pr}[s_{t}|s_{t-1}]$, where $s_{t-1},s_{t}\in S$ and *t* = 2,3,…, *T* are the time steps. Here, a time step is defined as every separate box opening/fixation. The attentional states correspond to the payoffs and probabilities in the binary risky choice in each trial.

The central assumption of our model is that attentional transition probabilities depend on the attribute value revealed by the attentional state (figure 1*b*). Specifically, attending to a high probability increases the likelihood of subsequently attending to the payoff of the same branch and attending to a high payoff increases the likelihood of subsequently attending to the corresponding branch probability. Thus, for an attentional state *s_t_* that belongs to the same branch as $s_{t-1}$, $\mathrm{Pr}[s_{t}|s_{t-1}]$ is increasing in $z_{t-1}$, the (normalized) probability or payoff revealed at the previous attentional state $s_{t-1}$.

In our main Markov attention model, we allow both probability-to-payoff interactions (according to which high probabilities direct attention to payoffs in the same branch) and payoff-to-probability interactions (according to which high payoffs direct attention to probabilities in the same branch), and thus call it the *dual-interaction* model. Formally, we write $\mathrm{Pr}[s_{t}|s_{t-1}]$, as a function of three sets of predictor variables:

2.1 $$\mathrm{Pr}[s_{t}|s_{t-1}]=\sigma \left(\;\begin{array}{c} \beta_{1}B_{t}^{\mathrm{Optn}}+\beta_{2}B_{t}^{\mathrm{Attr}}+\beta_{3}B_{t}^{\mathrm{Bran}}+\;\\ \beta_{4}I^{\mathrm{Optn}}+\beta_{5}I^{\mathrm{Attr}}+\beta_{6}I^{\mathrm{Stat}}+\beta_{7}I^{\;p\to \mathrm{Bran}}+\beta_{8}I^{I^{x\to \mathrm{Bran}}}+\;\\ (\beta_{9}I^{\;p\to \mathrm{Bran}}+\beta_{10}I^{I^{x\to \mathrm{Bran}}})z_{t-1},\; \end{array}\;\right),$$

where σ(⋅) is the softmax function such that $\underset{s_{t}\in S}{\sum }\mathrm{Pr}[s_{t}|s_{t-1}]=1$. For expositional convenience, we use $\beta_{\mathrm{prob}-\mathrm{to}-\mathrm{pay}}$ and $\beta_{9}$ interchangeably, and use $\beta_{\mathrm{pay}-\mathrm{to}-\mathrm{prob}}$ and $\beta_{10}$ interchangeably in the paper.

Line 1 of the right-hand side of equation (2.1) denotes a set of bias predictors. $B_{t}^{\mathrm{Optn}}$ specifies whether *s_t_* belongs to the option on the left or right side of the screen (1 if left; 0 if right), $B_{t}^{\mathrm{Attr}}$ specifies whether *s_t_* belongs to the payoff attribute or the probability attribute (1 if payoff; 0 if probability), and $B_{t}^{\mathrm{Bran}}$specifies which branch *s_t_* belongs to (for two-branch experiments, 1 if the first branch; 0 if the second branch; for five-branch experiments, the first, second, …, fifth branches are represented by 1, 0.75, 0.5, 0.25, 0, respectively. This coding assumes that a branch-wise attentional bias linearly increases/decreases from top to down in the five-branch experiments.). Therefore, $\beta_{1}$, $\;\beta_{2}$ and$\;\beta_{3}$ describe option-wise, attribute-wise and branch-wise attentional biases, respectively.

Line 2 captures a set of transition predictors. $I^{\mathrm{Optn}}$ indicates whether *s_t_* and $s_{t-1}$ belong to the same option (1 if same; 0 if different), $I^{\mathrm{Attr}}$ indicates whether *s_t_* and $s_{t-1}$ belong to the same attribute (1 if same; 0 if different) and $I^{\mathrm{Stat}}$ indicates whether *s_t_* and $s_{t-1}$belong to the same state (1 if same state; 0 if different). $I^{\;p\to \mathrm{Bran}}$ indicates whether $s_{t-1}$ is a probability attribute and *s_t_* and $s_{t-1}$ belong to the same branch, whereas $I^{x\to \mathrm{Bran}}$ indicates whether $s_{t-1}$ is a payoff attribute and *s_t_* and $s_{t-1}$ belong to the same branch (1 if true, 0 if false). For expositional brevity, we drop the subscripts *t* and *t*-1 in these variables. Therefore, $\beta_{4}$, $\;\beta_{5}$ and$\;\beta_{6}$ describe within-option, within-attribute and within-state transition tendencies, respectively. $\beta_{7}$ describes within-branch transition tendencies starting from a probability attribute and $\beta_{8}$ describes within-branch transition tendencies starting from a payoff attribute. Note that although we did not record consecutive resampling of the same state in the two five-branch experiments, we kept $\beta_{6}$ in the model to be consistent with other experiments. As shown in electronic supplementary material, table S4, the estimated $\beta_{6}$ are substantially negative in all the experiments, particularly in the two five-branch experiments.

Finally, line 3 captures the set of dynamic transition predictors that capture interactive attention. Here, $z_{t-1}$ represents the observed attribute value in $s_{t-1}$, the most recent attentional state. To make the estimated parameters comparable across attributes, we standardized the values along each attribute (probability or payoff) using *z*-scoring. Therefore, $\beta_{9}$ (which is $\beta_{\mathrm{prob}-\mathrm{to}-\mathrm{pay}}$ in the subsequent text) describes the extent to which the value of a branch probability increases the likelihood of transitioning to its associated branch payoff (a probability-to-payoff interaction), while $\beta_{10}$ (which is $\beta_{\mathrm{pay}-\mathrm{to}-\mathrm{prob}}$ in the subsequent text) describes the extent to which the value of a branch payoff increases the likelihood of transitioning to its associated branch probability (a payoff-to-probability interaction).

We also specified starting point probabilities using:

2.2 $$\mathrm{Pr}[s_{1}]=\sigma (\beta_{1}x_{t}^{\mathrm{Optn}}+\beta_{2}x_{t}^{\mathrm{Attr}}+\beta_{3}x_{t}^{\mathrm{Bran}}),$$

where *s*_0_ represents the start of a trial and σ(⋅) is the softmax function that sets $\sum_{s_{1}\in S}\mathrm{Pr}[s_{1}]=1$. This specification allows the first searched attentional state $s_{1}$ in each trial to depend on option-wise, attribute-wise and branch-wise attentional biases $\beta_{1}$, $\;\beta_{2}$ and $\;\beta_{3}$.

In addition, we fit three additional Markov attention models to evaluate the importance of each of the interaction parameters. The probability-interaction model turned off the payoff-to-probability interaction parameter (by setting $\beta_{9}=\;\beta_{\mathrm{pay}-\mathrm{to}-\mathrm{prob}}=0$), the payoff-interaction model turned off the probability-to-payoff interaction parameter by setting $\beta_{10}=\;\beta_{\mathrm{prob}-\mathrm{to}-\mathrm{pay}}=0$). The baseline model turned off both interactions (by setting $\beta_{9}=\beta_{\mathrm{prob}-\mathrm{to}-\mathrm{pay}}=\beta_{10}=\beta_{\mathrm{pay}-\mathrm{to}-\mathrm{prob}}=0$) and thus assumed that attention does not depend on the specific probability and payoff values observed in the previous attentional state.

We used hierarchical Bayesian analysis to fit the Markov attention models, and to generate the predicted transitions and attention proportions (see electronic supplementary material, Methods for details)**.**

Note that, for the tractability in predicting attention proportions, we assumed that the attention process during decision making was a Markov process. This was a strong assumption because the participants were likely to hold more than one piece of information in memory when making subsequent information search. In a supplementary attention model, we allowed the decision maker to keep a record of all searched information and tested whether they were more (or less) likely to attend to unsearched information, in addition to all the predictors in the dual-interaction attention model. Mathematically, the supplementary attention model becomes:

$$\mathrm{Pr}[s_{t}|s_{t-1}]=\sigma \left(\;\begin{array}{c} \beta_{1}B_{t}^{\mathrm{Optn}}+\beta_{2}B_{t}^{\mathrm{Attr}}+\beta_{3}B_{t}^{\mathrm{Bran}}+\;\\ \beta_{4}I^{\mathrm{Optn}}+\beta_{5}I^{\mathrm{Attr}}+\beta_{6}I^{\mathrm{Stat}}+\beta_{7}I^{\;p\to \mathrm{Bran}}+\beta_{8}I^{I^{x\to \mathrm{Bran}}}+\;\\ (\beta_{9}I^{\;p\to \mathrm{Bran}}+\beta_{10}I^{I^{x\to \mathrm{Bran}}})z_{t-1}+\;\\ \beta_{11}I_{t}^{\mathrm{Novelty}}\; \end{array}\;\right),$$

where $I_{t}^{\mathrm{Novelty}}$, indicates whether the attentional state, *s_t_*, had been previously searched in the trial (1 = unsearched, 0 = searched). Thus $\beta_{11}$ captures the tendency to sample novel pieces of information in the attention process. This analysis suggests that the participants displayed strong tendencies to sample unsearched information, violating the Markov assumption. However, this extension did not change all the key findings based on the dual-interaction attention model (see electronic supplementary material, tables S4 and S5). Thus, our main analysis was based on the dual-interaction attention model with the Markov assumption.

### (c) Attentional drift diffusion choice models

To evaluate the role of attention (both empirical and predicted) in predicting choices and response times, we examined a drift diffusion model [8,9] with the assumption that the drift rate depends on the amount of attention to the underlying payoffs and probabilities of the two options, as with the attentional drift diffusion model (ADDM). In standard drift diffusion models, preferences for or against the two options are accumulated up to a boundary, and the mean rate of this accumulation is captured by the drift rate. In the ADDM, this drift rate further depends on the information that is attended to during the trial (figure 1*c*). In our implementation of ADDM, we assumed that higher attention to the payoffs or probabilities of an option lead to a higher weight on these payoffs or probabilities in the drift rate. As with other drift diffusion models, choices were made when a positive or negative threshold was crossed [10,23]. In the ADDM choice models, the drift rate *v* expresses as a relative preference for *X* over *Y*. Here, we write the drift rate as a function of unweighted payoffs, attention-weighted payoffs and attention-weighted probabilities:

2.3 $$v=\alpha +u_{\mathrm{pay}}\left(\frac{1}{N}\overset{N}{\underset{n=1}{\sum }}x_{n}-\frac{1}{M}\overset{M}{\underset{m=1}{\sum }}y_{m}\right)+w_{\mathrm{pay}}\left(\frac{1}{N}\overset{N}{\underset{n=1}{\sum }}\bar{a_{x_{n}}}\cdot x_{n}-\frac{1}{M}\overset{M}{\underset{m=1}{\sum }}\bar{a_{y_{m}}}\cdot y_{m}\right)+w_{\mathrm{prob}}\left(\frac{1}{N}\overset{N}{\underset{n=1}{\sum }}\bar{a_{\;p_{n}}}\cdot p_{n}-\frac{1}{M}\overset{M}{\underset{m=1}{\sum }}\bar{a_{q_{m}}}\cdot q_{m}\right),$$

where $\bar{a_{s}}\;\;(s\in S=\{x_{n},p_{n},y_{m},q_{m}|n=1,2,\ldots ,N;m=1,2,\ldots ,M\;\})$ is the attention proportion to the attentional state *s*. *N* and *M* indicate the number of branches in *X* and *Y*, respectively. $x_{n}$ and $p_{n}$ denote the *n*th payoff and probability, respectively, in *X*. $y_{m}$ and $q_{m}$ denote the *m*th payoff and probability, respectively, in *Y*. α is the intercept in the drift rate. *u*_pay_ determines the effect of unweighted average of payoffs on the drift rate, regardless of attention. In other words, *u*_pay_ determines the accumulation of unattended payoffs. *u*_pay_ = 0 implies that the unattended payoffs are not accumulated in the decision process. In the MouseLab experiments, an unseen branch payoff is replaced with zero. *w*_pay_ determines the *additional* accumulation bias for payoffs due to attention. The accumulation of attended payoffs is jointly determined by *u*_pay_ and *w*_pay_. *w*_prob_ determines the accumulation bias for probabilities due to attention. Note that the unweighted average of probabilities is not in the equation because *N* = *M* in all the experiments and thus the corresponding term $(1/N)\sum_{n=1}^{N}\;p_{n}-(1/M)\sum_{m=1}^{M}q_{m}$ always equals 0.

Depending on the way in which the attention proportion $\bar{a_{s}}$ was generated, we had five variants of the ADDM choice models. Primarily, empirical-ADDM directly used the attention proportions, $\bar{a_{s}}$, from the empirical data. We also tested another four ADDM models based on the predicted attention proportions by each of the four Markov attention models, respectively. Dual-ADDM used the predicted attention proportions by the dual-interaction attention model, probability-ADDM used those by the probability-interaction attention model, payoff-ADDM used those by the payoff-interaction attention model and baseline-ADDM used those by the baseline attention model. Note that since empirical-ADDM used the more accurate attention data, its fits should be better than those of other ADDM models.

In the DDM choice model that does not take into account the attention allocated to the different objects, the drift rate is simply

2.4 $$v=\alpha +u_{\mathrm{pay}}\left(\frac{1}{N}\overset{N}{\underset{n=1}{\sum }}x_{n}-\frac{1}{M}\overset{M}{\underset{m=1}{\sum }}y_{m}\right),$$

It is interesting to note that the DDM choice model as specified above is equivalent to the equiprobable heuristic, one that ignores the probability attribute for risky decision making, on the choice side.

We fit the ADDM and DDM choice models using hierarchical Bayesian analysis (see electronic supplementary material, Methods for details). Note that the ADDM model used here is not, strictly speaking, the original ADDM model [8,9]. In the original ADDM, the drift rate changes after each attention sample. By contrast, we have simplified the drift rate in our model to be a time invariant average of the attention-weighted attributes. Our simplification gives us considerable computational tractability and allows us to use hierarchical Bayesian methods for model fitting. It also does not detract from the key contribution of this paper, which has to do with modelling attention dynamics. Lombardi and Hare have performed a rigorous analysis of the relationship between the type of attention-weighted model investigated in this paper and the more complex time varying drift rate model used in other applications and have shown that the two are very similar when the drift rate in the former model is a function of the (known) reaction time and relative attention proportion to the attributes [47].

### (d) Dominance violation study

We ran an additional one-item risky choice experiment involving dominance with the students in an undergraduate class at a private university in North America (*N* = 205). The choice was between *X* = {$100, 0.99; $1, 0.01} and *Y* = {$100, 0.01; $1, 0.99}. Subjects were asked to indicate their preference in a binary choice.

## 3. Results

### (a) Summary of data

As mentioned above, we conducted two new five-branch risky choice experiments with the MouseLab paradigm. We also reanalysed mouse-tracking and eye-tracking data from four existing two-branch risky choice experiments. Overall, our full analyses involved 22 285 choices from 340 participants in six experiments, including eye-tracking data from 2825 choices and 57 participants, and mouse-tracking data from 19 460 choices and 283 participants.

In our two five-branch experiments, participants chose the EV-maximizing gamble in 77% and 73% of trials. Similarly, in Pachur *et al*.'s [27] two mouse-tracking experiments, EV-maximization rates were 68% and 69%, respectively. Participants in the study by Fiedler & Glöckner [29] had an EV-maximization rate of 53% in exp. 1 and 67% in exp. 2. Across the six experiments, participants obtained an average of between 21 and 36 attentional samples (eye movements or mouse clicks) per trial (see electronic supplementary material, table S6 for other details).

### (b) Strong interactive attention effects in all datasets

**Figure 2.**
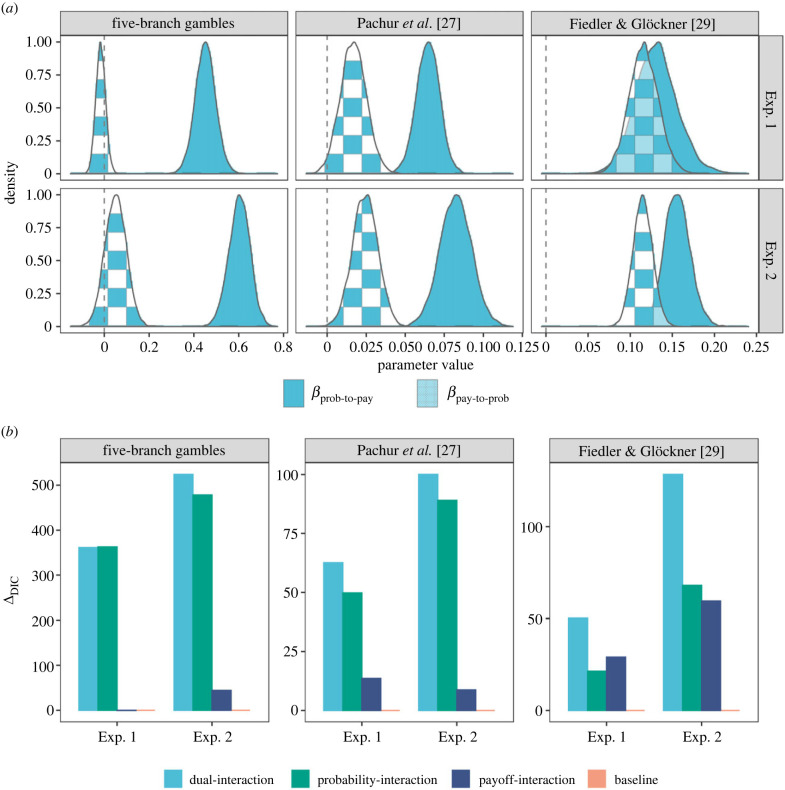
Hierarchical Bayesian fits of the attention models. (*a*) Posterior distribution of group-level interactive attention parameters in the dual-interaction attention model. (*b*) Deviance information criterion difference (*Δ*_DIC_) between each of the attention models and the baseline attention model that does not allow for interactive attention. The higher the *Δ*_DIC_ value, the better the model fit. The *Δ*_DIC_ values for the baseline attention model are always 0. Positive values of *Δ*_DIC_ for our models provide strong evidence for interactive attention in all experiments.

We fit our Markov attention models to data from the six experiments. In all cases, we found that the group-level *β*_prob-to-pay_ coefficients were significantly positive, with all 95% credible intervals higher than zero (figure 2*a*), showing that there are strong interactive attention effects both in mouse-tracking data and in eye-tracking data, and both in two-branch and in five-branch gambles. Our fits also revealed that the *β*_prob-to-pay_ coefficients were larger than the corresponding *β*_pay-to-prob_ coefficients in nearly all experiments. This contrast is especially compelling in the four mouse-tracking experiments (with all 95% credible intervals much higher than zero, see electronic supplementary material, table S4 for details). In the two eye-tracking experiments, however, a slightly more balanced interactive attention pattern in the two directions was observed (with 95% credible intervals close to or including zero). It is also worth noting that the five-branch gamble experiments typically have much larger interactive attention coefficients than the two-branch gamble experiments, indicating that the interactive attention may play a stronger role in settings with more complex information structures.

To more formally examine the relative fits of our models, we carried out a model comparison analysis using the deviance information criterion (DIC) (figure 2*b*). This analysis revealed that the three interactive attention models fit the attention data better than the baseline model, providing further evidence for interactive attention. We also found that the dual-interaction and the probability-interaction models were always better than the payoff-interaction model. The only exception was exp. 1 of Fiedler & Glöckner [29], where the probability-interaction model lagged slightly behind the payoff-interaction model, with a DIC margin of 7.71. While the payoff-interaction model performed poorly, the probability-interaction model performed almost as well as the dual-interaction model, especially in the mouse-tracking experiments. This once again indicates that attending to high probabilities drives subsequent attention to branch payoffs, but that the converse effect is weaker. This is also consistent with prior theoretical work that proposes that attention to payoffs depends on probabilities [11,26,42], but not vice versa.

### (c) Markov attention models predict empirical patterns

**Figure 3.**
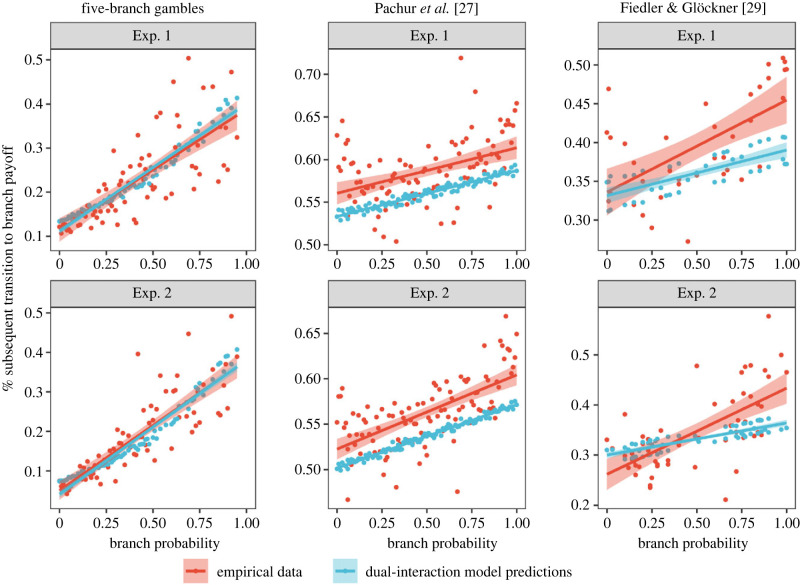
Probability-to-payoff interactive attention patterns and model predictions. Each point corresponds to a single gamble branch. The *x*-axis represents the probability in the branch while the *y*-axis represents the proportion of transitions to the associated payoff in the same branch immediately after observing the branch probability. The empirical data averages across all individuals. The predictions of the dual-interaction model are the average of individual-level predictions generated by the full set of individual-level posterior parameters. The positive correlations in this figure provide qualitative evidence for interactive attention in all six experiments.

Figure 3 provides qualitative evidence for interactive attention, by showing how attention to high or low branch probabilities influences the subsequent attentional sample, in our empirical data. Figure 3 also illustrates our dual-interaction model's predictions for this effect with high accuracy (see electronic supplementary material, figure S2 for a predicted versus observed visualization).

As can be seen, attending to a high (low) probability value increased (decreased) the likelihood that participants sampled a payoff from the same branch of the gamble in the successive attention state. This effect is strong in all six datasets, as indicated by positive group-level correlations between branch probabilities and the proportion of subsequent transitions to the payoff in the same branch (all *r* ≥ 0.43, all *p* < 0.001). As expected, this pattern can be predicted by the dual-interaction model, whose dynamics closely resemble those observed in the data, and generate strong positive correlations between observed branch probabilities and the predicted proportion of subsequent transitions to the payoff in the same branch (all *r* ≥ 0.76, all *p* < 0.001). This interactive attention dynamics has led to higher global attention proportions to the payoffs associated with high probabilities (see electronic supplementary material, figure S3). In electronic supplementary material, figure S4, we show the converse pattern (observing a high payoff increases the likelihood that participants sample the corresponding branch probability in the successive attention state). This pattern is weaker than the pattern shown in figure 3, which explains the somewhat worse fits of the payoff-interaction attention model in figure 2*b*. In electronic supplementary material, Results, we also show that the Markov attention models, especially the dual-interaction attention model, accurately capture both individual-level and trial-level heterogeneity.

**Figure 4.**
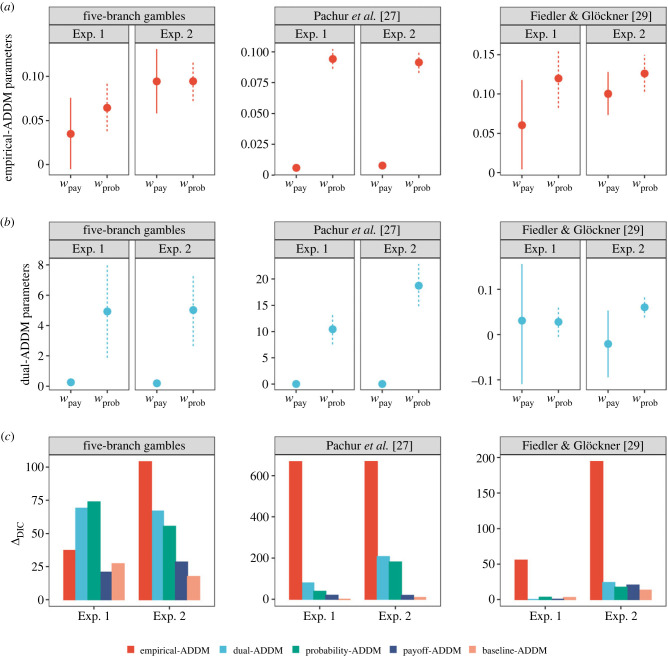
Parameter estimation and DIC values of main attentional drift-diffusion models (ADDM). (*a*) Bayesian estimation of the empirical-ADDM model parameters (posterior means and 95% credible intervals). (*b*) Bayesian estimation of the dual-ADDM model parameters (posterior means and 95% credible intervals). (*c*) Relative model fits (*Δ*_DIC_) between different versions of the ADDM models and the DDM model that does not consider attention proportions when predicting choice and response time. The larger the *Δ*_DIC_ values, the better the ADDM model's performance. Positive parameter values and positive *Δ*_DIC_ values indicate that both empirical and model-generated attention proportions improve the prediction of choice and response time data.

### (d) Markov attention models improve choice and response time predictions

The above sections have shown that participants display interactive attention and that our Markov attention models successfully describe attentional dynamics on the group level, as well as on the individual level and the trial level. In this section, we test whether equipping the prominent attentional drift-diffusion model (ADDM) with either empirical attention data, or the predicted attention data from our Markov attention models, improves its fits to choices and response times. Figure 4*a* displays the Bayesian estimation of the attention weights for payoffs (*w*_pay_) and probabilities (*w*_prob_) at the group level for the main ADDM models. As can be seen in this figure, *w*_pay_ and *w*_prob_ are always positive in the *empirical-ADDM* model in all experiments, suggesting that the amount of attention to payoffs and probabilities positively predicts choice and response time in these experiments. In *dual-ADDM*, the choice model that uses predicted attention proportions from the main dual-interaction attention model, the attentions weights for payoffs (*w*_pay_) and probabilities (*w*_prob_) are almost always positive. The exception to this is exp. 1 of Fiedler & Glöckner [29], for which the two group-level parameters, *w*_pay_ and *w*_prob_, although positive, include zero in the 95% credible intervals. In exp. 2, *w*_pay_ is also not different from zero (see electronic supplementary material, table S7 for other parameter values).

A model comparison using DIC further demonstrates that both empirical attention data and model-generated attention data improve predictions of choice and response time. Figure 4*c* shows that almost all the ADDM models outperform the simple DDM model, the latter of which does not take attention data into account (see equation (2.4)). The exception to this is exp. 1 of Fiedler & Glöckner [29], for which the ADDM models with the predicted attention input do not outperform DDM. A further comparison between the four prediction-based ADDM models suggests that the choices and response times are better explained if the interactive attention parameters (especially $\beta_{\mathrm{prob}-\mathrm{to}-\mathrm{pay}}$) are specified in the attention model. In particular, the dual-ADDM and the probability-ADDM models always predict choices and response times better than the payoff-ADDM and the baseline-ADDM models do. Posterior predictive check suggests that the dual-ADDM is able to predict actual choice proportions at the trial level in all experiments at a moderate level (see electronic supplementary material, figure S5), although it does not predict well the actual response times at the trial level, especially in the MouseLab experiments (see electronic supplementary material, figure S6). It is worthwhile noting that, in predicting both choice and response time, the dual-ADDM model significantly underpredict the variation across trials. Such predictive losses suggest that our dual-ADDM may be limited in capturing the full attention, response time and choice patterns, as discussed below.

The empirical-ADDM model, which uses actual attention proportions, outperforms the ADDM models that use our Markov attention models' predicted attention proportions as input (with the exception of the five-branch-gambles in exp. 1, where dual-ADDM and probability-ADDM outperform empirical-ADDM). This suggests that, other than interactive attention dynamics, there are additional attentional mechanisms that guide sequential evidence accumulation for decision making. Previous research has suggested that the attention during the decision process may be directed to visually salient objects [48], and options with high values [49,50] or options with uncertain values [51,52]. Such attention processes may also play a crucial role in predicting choice and response time, though they are not the focus of the present paper. Of course, it may also be possible that unsystematic noise, not captured by our model, is responsible for the difference between the empirical-ADDM and dual-ADDM. Future research should attempt to model other determinants of attention and thus disentangle systematic sources of attentional bias from unsystematic noise. This somewhat inevitable inaccuracy in attention predictions may have modified the parameter values estimated from the ADDM models. As can be seen in figure 4*a*,*b*, the estimated parameter values in dual-ADDM appear to be larger than those in empirical-ADDM. That is likely because our dual-interaction attention model, while generating attention proportions highly correlated with the empirical attention proportions (see electronic supplementary material, Results), underpredicts the variability of attention proportions allocated to different attributes in the experiments. This causes the predictions to cluster near the centre of the distribution, reducing their range and causing estimated parameters to be larger.

Despite the superior fits of empirical-ADDM, there are many reasons why our approach is beneficial. Firstly, eye gaze data is not always available. Thus, if the goal is to predict choice, we need a model of attentional dynamics, such as dual-ADDM. Secondly, a complete theory of the choice process needs to specify both how attention guides the formation of preferences, as well as how the choice set guides the allocation of attention [49,51]. Empirical ADDM addresses the first goal but not the second. In this way, dual-ADDM fills in a large theoretical gap in the literature. Foremost, by modelling the allocation of attention, dual-ADDM helps solve a related theoretical problem: how psychologically realistic choice processes can generate multiplicative economic utility functions.

### (e) Interactive attention is necessary to make reasonable choice predictions

Finally, let us return to the example problem presented in the introduction: the choice between *X* offering a 99% chance of $100 and a 1% chance of $1, versus *Y* offering a 1% chance of $100 and 99% chance of $1. *X* strongly dominates *Y*, and all SEUT models predict that *X* will be chosen over *Y*. Simple additive accumulation models without interactive attention, however, cannot distinguish *X* from *Y* as both offer $100 and $1 as payoffs and 99% and 1% as probabilities.

To test if people can successfully avoid such dominance violations, we ran a study asking 205 participants to choose between *X* and *Y*. We found that 201 participants chose *X* over *Y* (*p* < 0.001 in a binomial test), in line with the predictions of all SEUT models and in contradiction to the predictions of all additive models without interactive attention.

Can our dual-interaction attention model, combined with the ADDM, explain this effect? To test this, we used the posterior samples of individual-level parameters in our attention models from our six experiments, to generate *out-of-sample* predictions of the attention proportions during a choice between *X* and *Y*. The predicted attention proportions were in turn used to calculate the drift rate favouring *X* over *Y* (see electronic supplementary material, Methods for details)*.* We found that 100% of the participants in our six experiments had a drift rate favouring *X* over *Y*, showing that our main dual-interaction attention model could, like our human participants, successfully avoid dominance violations in risky choice. By contrast, the baseline attention model, which does not have interactive attention, predicted a drift rate of zero, indicating that it was indifferent between *X* and. Thus, interactive attention is necessary to make reasonable predictions in risky choice. See electronic supplementary material, figure S7 for the predictions of other Markov attention models for the choice between *X* and *Y*.

## 4. Discussion

The goal of this paper has been to relate cognitive and neurocognitive models of decision making to those commonly studied in the economic and social sciences. This has been the focus of considerable interdisciplinary interest, but progress has been difficult. Prominent cognitive and neurocognitive models assume that preferences accumulate in additive increments, guided by attention to the individual attributes of choice options [8,10,23]. Yet in order to make good decisions, and to match the predictions of economic theories, the attributes of options often need to be aggregated in a multiplicative or interactive manner. In risky choice, for example, SEUT models assume that payoffs are multiplied against corresponding probabilities, allowing the decision maker to successfully evaluate multi-branch gambles and avoid simple mistakes involving transparent dominance [1,2,4]. Standard accumulation models, which weigh and sum up each piece of sampled information separately, cannot avoid these mistakes and are thus not typically applied to risky choice.

Note that one could, in principle, generate an accumulation model in which the drift rate was simply the difference between two nonlinear utility functions (e.g. the difference between the EVs, expected utilities, or prospect theory utilities of the gambles). However, such a model would not constitute a complete mechanistic account of the decision process, as it does not specify the representational and algorithmic elements necessary to compute nonlinear utilities, and does not relate the process of nonlinear utility formation to the (sequential) attention samples that underlie information acquisition. Standard accumulation models, by contrast, do provide a complete mechanistic account as sequentially sampled information is sequentially added to a running accumulator variable. Such models do quite well in low-level perceptual decision making (in which additive operations are suitable for aggregating sequentially obtained information), and even in some value-based decision making settings that have this structure. However, they cannot provide a good account of multi-branch risky choice, in which attribute values interact.

We have examined one solution to this problem. According to our proposal, attention to high payoffs or probabilities increases subsequent attention to payoffs and probabilities within the same branch of the gamble. Thus, even though the accumulation process combines inputs additively, interactive attentional dynamics imply that the rate of accumulation is a multiplicative function of the gamble probabilities and payoffs, mimicking the desirable normative and descriptive properties of SEUT. We have formalized our interactive attention mechanism in a Markov model, combined it with a standard accumulator, and fit the resulting model on four existing and two new eye-tracking and mouse-tracking datasets. We have found strong evidence for interactive attention in all of the datasets, and have shown that our model predicts key patterns in attention and choice. In doing so, we have introduced a number of technical and theoretical innovations to the modelling of decision making. For example, ours is one of the first quantitative models that can be jointly fit to attention, choice and response time data in complex risky decisions.

By directly relating established theories in psychology and neuroscience to those in economics, our results open up a number of useful research directions at the interface of the social and natural sciences. For example, future work could use our modelling framework to relate attention to other interesting properties of economic models of risk taking, including properties like loss aversion and probability weighting [15,27,53]. Future work could also try to understand the economic implications of the empirical patterns documented in this paper (such as the stronger interactive attention effect of probabilities on payoffs, rather than vice versa). It would also be useful to test the robustness of these patterns, and the interactive attention mechanism more generally, in real-world economic settings. For example, it could be the case that our model is less relevant for settings in which the probability and payoff for a particular branch of the gamble can be sampled simultaneously. Finally, by parametrizing participant-level heterogeneity in interactive attention and decision making, our approach also allows researchers to relate individual-specific cognitive and neural variables to individual-specific economic and social outcomes, facilitating a more rigorous analysis of behaviour across diverse groups [54–59]. Testing the feasibility of this approach is a very promising topic for future work.

Of course, our core insight extends beyond risky choice. Multiplicative utility functions are the basis of economic decisions in intertemporal, social, consumer and strategic choice [3,5,6,13]. Accumulation processes have been shown to describe choice behaviour in these domains [26,60–66], and it is likely that interactive attention is involved in the aggregation of information to generate interactive utility [43,44,67–71]. Thus, better understanding interactive attention will not only further basic research on different facets of choice behaviour, but will also facilitate novel practical and policy-relevant applications, including those pertaining to health, financial, social, managerial and consumer decision making [61,72,73].

It is important to note that our paper, as well as the rich literature that motivates it, relies on the implicit assumption that attention determines the formation of preferences. This is an assumption that has been criticized [74], and it is possible that the link between attention and preference has the opposite causal direction, that is, that decision makers are more likely to look at preferred options [49,50]. Moreover, the interplay between attention and choice may be driven by normative concerns, like the resolution of uncertainty and the selection of the most desirable option with the least amount of cognitive effort [46,49,51,52]. Our work contributes to the study of the relationship between attention and choice in two important ways. First, by allowing for high-valued attributes to bias attention, and for attention, in turn, to bias the formation of preference, our approach can help disentangle the causal link between attention and preference in a model-based manner. Second, we show that the bidirectional interplay between attention and choice is necessary for mimicking the properties of normative economic models like EV and expected utility maximization. This in turn is necessary for making good choices (avoiding dominance violations) when offered multi-branch gambles. Future work could attempt to relate this economic argument with the statistical arguments discussed above. Overall, we look forward to modifications and extensions of our framework that integrate multiple cognitive mechanisms, along with diverse perspectives from economics, psychology and neuroscience, in order to develop a unified quantitative model of human choice behaviour.

## Data Availability

All code and data are publicly available in an OSF repository: https://osf.io/9kzm3/. The data are provided in electronic supplementary material [75].
